# Technological Innovation and Trade Orientation as Drivers of Renewable Energy Consumption: Panel Evidence from G7 Economies

**DOI:** 10.1002/gch2.202400345

**Published:** 2025-01-29

**Authors:** Minyan Yu, Umar Farooq, Mosab I. Tabash, Abdullah A. Aljughaiman, Abdulateif A. Almulhim

**Affiliations:** ^1^ Jiangxi Modern Polytechnic College Nanchang 330095 China; ^2^ School of Economics and Finance Xi'an Jiaotong University Xi'an Shaanxi Province 710049 P. R. China; ^3^ College of Business Al Ain University Al Ain 64141 United Arab Emirates; ^4^ Finance Department School of Business King Faisal university Al hafouf 31982 Saudi Arabia; ^5^ Finance Department Business School king Faisal University Al hafouf 31982 Saudi Arabia

**Keywords:** export orientation, import orientation, renewable energy, technological innovation, trade orientation

## Abstract

Recent advancements in industrialization have sparked substantial interest among the academic community and policymakers regarding energy consumption. The consumption patterns of distinct energy types seem to be influenced by many national and international factors. In this essence, this study investigates the effects of technological innovation and trade orientation on renewable energy consumption (REC). Using panel data from G‐7 economies from 2000 to 2019, Panel EGLS (estimated generalized least square) and ARDL (autoregressive distributed lag) models are employed to check the regression. The analysis reveals that both technological innovation and export orientation have a significant and positive impact on REC. In contrast, import orientation negatively affects REC. The results highlight that technological advancements and a focus on export‐oriented trade strategies can significantly boost REC. Policymakers are encouraged to invest in research and development to advance renewable energy technologies and provide incentives for their adoption. These measures are crucial for achieving environmental sustainability and reducing pollution. The study provides valuable insights into how national and international trade dynamics influence renewable energy usage.

## Graphical abstract

Technological innovation and trade orientation significantly influence renewable energy consumption in G7 economies. Using data from 2000 to 2019, the study reveals that technological advancements and export orientation positively impact renewable energy use, while import orientation has a negative effect. These findings underscore the importance of fostering innovation and export‐driven policies to achieve environmental sustainability and promote clean energy adoption.

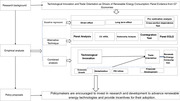

## 1 Introduction

The massive expansion in economic activities and population expansion has driven the global energy demand to rise to an alarming level.^[^ ^1^, ^2^ ^]^ It is the economic powerhouse of developed and developing countries. A constant energy supply is crucial to meeting the energy needs for sustainable economic growth.^[^ ^3^ ^]^ Increasing energy usage boosts the economy but may cause environmental damage. Consequently, temperatures rose 0.4 °C to 0.8 °C in the last century, while CO_2_ secretions rose 31%. This concern is repeatedly debated and continues to rank among the biggest problems the world community is currently facing. Specifically, various policy activities aim to combat environmental problems and maintain global economic sustainability. Shifting from traditional energy sources to renewable alternative consumption (REC) is one way to control environmental hazards and to maintain the economic growth. Among others, technological innovation (TI) and trade focus can play vital role in driving the more REC. New technologies can now be widely used in the economy due to TI. TI can expedite the widespread adoption of REC, which can meet expanding energy demand, help energy providers to compete more, and has positive spillovers regarding low consumer prices. In this regard, trade increases the flow of goods and services, including high‐quality technical equipment and boosts aggregate productivity by reallocating resources and using inputs efficiently.^[^ ^4^, ^5^ ^]^ However, the industrial sector and the final domestic demand for imports in the G‐7 economies make a substantial contribution to CO2 emissions, indicating the use of nonrenewable energy, a significant trade driver. Although, most governments have comprehended the harmful effects of intensifying CO2 levels and are preparing for climate‐related dangers and extreme events. The COP conference serves as a forum for nations to engage in collective political deliberations around climate change. The present analysis seeks to determine the practical impact of TI and trade orientation on the consumption of renewable energy, based on theoretical arguments.

Renewable energy (sunlight, wind) is either inexhaustible or replenishable. Solar, wind, geothermal, hydropower, bioenergy, and ocean power are renewable energies.^[^ ^6^ ^]^ In power generation, heating and cooling, and transportation, renewables have increased. According to previous research, energy is an important component of industrial output. Economic growth cannot be attained without energy sources; as growth increases, so does energy usage. Although economists have created several theories on economic growth and development, these theories ignore energy as a crucial component. Similarly disregards the value of using renewable energy in this context. However, the Solow growth model considers TI crucial for economic growth. The sustainable development goals (SDG) are based on innovations, which are the foundation of technical advancement. Recently, TI has been included as a new measure in the growth‐environment framework because it's vital for reducing CO2 through REC worldwide. Using traditional and renewable energy sources efficiently requires TI. Innovation helps develop renewable energy sources. Technological advances increase the likelihood that renewable energy can meet future energy demand. Given the increasing demand for energy, it is anticipated that renewable energy, a sustainable and eco‐friendly form of energy, will become the primary source of energy.

Nevertheless, low‐cost, and large‐scale technological innovations drive renewable energy progress and energy transition.^[^ ^7^ ^]^ Trade is a macroeconomic characteristic that affects many economic indices. Diversifying trade products boosts total energy demand.^[^ ^8^ ^]^ Export‐oriented countries boost economic activity and domestic production. Rising domestic production will increase domestic energy demand. So, industries will fulfill the desired demand by consuming non‐renewable energy. However, under environmental pressure, export product diversification affects renewable energy usage as a structural change that changes the energy mix. In contrast, the import of technology that expands the utilization of renewable energy plants. This will enhance renewable energy use.^[^ ^9^ ^]^ Conversely, an economy may specialize in industrial products having polluting materials like cement and metal. These processes require a lot of energy to increase production and grow, which can be nonrenewable. Nevertheless, in the context of the economic situation of a country, trade patterns well define the REC.

Following the adoption of the Paris Climate Agreement (PCA) during the 2015 COP21 conference, all G‐7 countries have taken steps to transition to a sustainable, low‐carbon society. This includes diversifying their energy sources and prioritizing the utilization of renewable energy to promote environmental sustainability. However, the G‐7 economies have not yet succeeded in lowering CO2 secretions. These nations consume 30% and emit 25% of the world's energy and CO2 respectively.^[^ ^10^ ^]^ For our analysis, we have selected the group of seven (G‐7) nations for several reasons. First, the United States, Canada, the United Kingdom, Germany, Italy, France, and Japan, which make up the G‐7, are highly developed economies with large trade volumes and advanced technology. More than a third of global GDP originates from the G‐7. These countries control 58%, 35%, and 32% of global wealth, imports, and exports. Second, in relation to ongoing studies, these nations consume almost one‐third of the global energy supply and are the primary culprits behind carbon emissions.^[^ ^2^, ^11^ ^]^ Third, G‐7 nations rely heavily on imported and domestic nonrenewable energy. Most G‐7 nations import nonrenewable energy to meet their energy needs. For instance, Japan imports 96% of its primary energy, Italy 84%, and Germany 64%. These figures show the G‐7′s reliance on dirty fuels. These figures explain why these economically prosperous nations cannot manage environmental damage. However, the goal of the G‐7 is to use more renewable energy by 2050 in order to obtain net‐zero GHG secretions gas emissions. G‐7 countries can achieve the energy transition due to their economic strength. The higher TI helps these countries grow REC.

Innovation in technology can be a valuable tool for increasing the use of renewable energy sources, which the G‐7 industrialized economies need to do to meet their growing energy needs. Similarly, export‐oriented countries are concerned about product quality to increase exports. Moreover, export‐oriented countries can be able to transform their energy use into environmentally friendly energy. However, import‐oriented economies may not be able to manage their environmental standards. In this scenario, it is necessary to view how TI and trade orientation influence REC in G‐7 economies. The current study explores the effects of TI and trade orientation on REC in the G‐7 nations between 2000 and 2019. Globalization, FDI, GDP, and financial development are also included as controls in this study. The availability of the data dictated the time frame used. To correct for cross‐sectional dependence, we estimate the regression using the Panel EGLS model (estimated generalized least squares). We estimate the PMG/ARDL (pooled mean group) model for sensitivity analysis as most variables are stationary at level (1). Regarding EGLS and PMG/ARDL, the statistical analysis first demonstrates that TI has a significant positive influence on REC in the short and long term, respectively. In terms of trade, EXPO (IMPO) has a positive (negative) and significant impact on REC. This study documents GDP, FD, FDI, and GLOB as control variables. All control variables negatively influence the REC in the short term. However, FD and FDI positively correlate with REC in the long‐run.

Technological innovation plays a pivotal role in shaping REC by addressing key challenges associated with its adoption and efficiency. Advanced technologies enhance the efficiency of renewable energy systems, reduce production costs, and improve energy storage solutions, making renewables more accessible and reliable for widespread use. Moreover, innovations in smart grids and energy management systems optimize the integration of renewable energy into existing infrastructure, ensuring stable supply and demand alignment. Technological advancements also drive the development of new renewable energy sources, such as advanced biofuels and next‐generation solar panels, further diversifying the energy mix.^[^ ^1^ ^]^ Through these channels, technology not only accelerates the transition toward cleaner energy sources but also fosters energy security and environmental sustainability by mitigating reliance on conventional fossil fuels. Recognizing these pathways underscores the significance of investing in research and development to unlock the full potential of renewable energy.

This research makes three distinct contributions to the existing database of literature. Firstly, the work addresses a theoretical deficiency by incorporating trade orientation for a comprehensive perspective of the link between TI and REC in G‐7 countries. Given the limited number of studies conducted on the subject, the impact of trade orientation on the utilization of renewable energy within the G‐7 remains uncertain. Second, this work empirically adds, uses the PMG/ARDL and EGLS methodologies, and provides solid results. The explanatory variables' short‐ and long‐term effects taken into consideration on the REC are provided by the estimation of coefficients. The statistical study informs policymakers on the validity and importance of encouraging renewable energy through technical innovation. Third, empirical findings offer fundamental policies: According to this analysis, G‐7 firms must invest in renewable energy to meet their targets. Policymakers should promote R & D efforts by providing subsidies, direct funding, and moral backing. Accordingly, export orientation can be used as a tool for policy to increase REC. Increased dependency can be used to facilitate export enrichment, and export orientation can be used as a catalyst to advance the REC, which has numerous additional benefits in the form of environmental sustainability and economic development. G‐7 policy analysts should revisit the attached energy policies in light of economic expansion, financial advancement, international capital infusion, and globalization, even in the short term. These characteristics should be aligned with renewable energy use.

The organization of the study is as follows: Section 2 covers environmental literature on trade orientation, TI, REC, and G‐7 economies. Section 3 describes the data, models, estimating process, outcomes, and robust analysis. We present our findings in Section 4 in three sections: first, the preliminary analysis; second, the panel regression analysis; and third, the robustness check. The detailed discussion is presented in Section 5. Section 6 summarizes research evidence and policy implications.

## 2 Review of Literature

This section mainly focuses on building the link between explained and explanatory variables by exploring prior literature. In addition, we also build directional hypotheses based on previous literature in this section. Previous literature lacks substantial investigations regarding the liaison between trade orientation and technological innovation (TI) on the utilization of renewable energy. The connection of explanatory variables with explained variable is following as:

In this contemporary time, achievers adopt smartness. An advancement in technology brings down the cost and enhances the efficiency. The TI strongly hits economies with huge emissions of CO2, which resultantly produce renewable energy at minimum cost at greater energy efficiency as well. The shift from conventional to sustainable energy resources is aided by the expansion of the economy.^[^ ^1^, ^12^, ^13^ ^]^ The technological newness affects renewable sources of energy positively and significantly by using panel quantile regression augmented.^[^ ^14^ ^]^ The study of Jin et al.^[^ ^15^ ^]^ suggested that TI brings positive links with renewable sources of energy. Some economies prefer innovation for economic sustainability and greater energy efficiency. It was confirmed that the TI would provide a substantial short‐ and long‐term contribution to improving energy effectiveness.^[^ ^16^ ^]^ Another study done by Saudi et al.^[^ ^17^ ^]^ used three measurements of TI, e.g., investment in research and development (R&D), patents obtained by domestic entities, and the exportation of technologically advanced products. Their study additionally revealed that foreign direct investment (FDI) has a detrimental effect on the adoption of renewable energy within the context of Indonesia. The study of Rafindadi^[^ ^18^ ^]^ noted that financial development boosts energy consumption while reducing CO2 emissions. Conversely, economic growth reduces energy consumption but leads to higher CO2 emissions. In short, TI brings smartness in machinery, which motivates towards renewable energy sources.

Trade strengths economy and economic operations but to sustain such operations, the energy sources have a vital importance. The nature of the economy matters for the usage of energy sources. Some economies prefer to nourish service industries and some focus on manufacturing industries. The focusing of economies on services industries are mostly import‐oriented economies and such economies have an inverse link between import and sustained energy sources in both short and long run. But manufacturing oriented economies consider export activities as their primary focus. The primary focus of such economies give priority to consuming fossil fuels to expand their business activities in the short and long run, they prefer to consume sustained energy sources. This reveals a negative connection between export activities and REC; however, it demonstrates a favorable link over an extended period. The work of Chen^[^ ^19^ ^]^ explored a positive link between per‐capita export and renewable utilization of energy by employing system GMM technique. Another work done by Jebli et al.^[^ ^20^ ^]^ proxied trade with per capita import and export and they further asserted that enhancement of import and export operations mitigate GHG gasses which means that there is a positive link between trade orientation and consumption of renewable energy. The study of Bento and Moutinho^[^ ^21^ ^]^ and Qing et al.^[^^]^ unveiled that international trade operations boost the release of hazardous gases (GHG) highlighting a negative link between trade and the sustained energy utilization sources. The increase in export activities favors the use of renewable energy sources, hence enhancing the ecological quality.^[^ ^22^ ^]^ The study of Rafindadi and Ozturk^[^ ^23^ ^]^ noted that there is no Granger causality between economic growth and natural gas consumption in context of Malaysia. Nevertheless, the data indicates that capital and exports contribute 29.18% and 41.46% to the country's GDP, respectively, subsequent to natural gas consumption levels of 20.04% and 42.29%. In brief, the activities regarding exports are inversely related to renewable energy sources in the short‐run but in the long‐run, the operations regarding export are directly related to renewable sources of energy.

The degree of advancement within the financial sector correspondingly bolsters other industries. Various levels of development decide the utilization of energy in three different effect channels: the wealth effect, the business effect, and direct effect.^[^ ^24^ ^]^ They further observed a negative link between the usage of renewable energy sources and the level of financial sector development. A provision of hefty funds urges businesses to expand the businesses and can acquire the benefit as much as they can. In most of the economies, the primary and easily available source of non‐renewable energy and this expedites the degree of CO2, but it discourages renewable sources of energy. The study of Chang^[^ ^25^ ^]^ discovered a negative link between renewable energy and progress in the finance industry which means that the availability of funds discourages renewable energy utilization in the short run. Another work of Topcu and Payne^[^ ^26^ ^]^ expresses that by taking utter benefits from funds encourages businesses to use fossil energy and discourages renewable energy which shows a negative relation between financial sector development and renewable energy sources. The research of Rafindadi^[^ ^27^ ^]^ found that the study's findings revealed the presence of an inverted‐U curve, indicating the existence of the Environmental Kuznets Curve (EKC) despite the declining income of the state. The above‐mentioned works unveil that the acquisition of funds give confidence and minimize the risk of the managers, which attract them towards the utilization of the non‐renewable energy sources and discourages REC.

The top priority of every economy is to bring improvement and development in the economy by using all resources. The economy uses both renewable and non‐renewable resources. The economic growth positively and negatively affects the REC sources. According to Eren et al.,^[^ ^28^ ^]^ the economic growth and financial sector development positively affected the usage of renewable sources of energy for the case of India by employing Dynamic Ordinary least Square (DOLS) during 1971–2015. They further suggested that the consumption of renewable energy sources and GDP growth are financial advancement driven in the long‐run and both have bidirectional causality in India. The study of Bekun et al.^[^ ^29^ ^]^ suggested that the REC source is negatively affected by economic growth. Another study of Dong et al.^[^ ^30^ ^]^ unveiled that the positive movement in economic growth encourages the managers to consume the renewable sources of energy which show that there is a positive link between economic growth and REC. The GDP growth makes individuals financially independent to consume both types of energy and this independence may bring positive or negative relationship between GDP growth and REC.

Foreign direct investment affects the utilization of renewable energy sources. It also affects the targeting country socially, politically, ecologically, and economically. The study of Khan et al.^[^ ^31^ ^]^ found the short‐run and long‐run impact of FDI and technological newness on renewable and non‐renewable energy sources. In addition, the statistical outcomes reveal that economic growth, FDI and technological newness is negatively linked to renewable energy utilization. Another study conducted by Ergun et al.^[^ ^32^ ^]^ found that FDI has been found to have a beneficial and substantial impact on the development and utilization of renewable energy sources between the years 1980 and 2013. The study of Khandker et al.^[^ ^33^ ^]^ posited that foreign direct investment (FDI) serves as a lucrative investment prospect that entices investors to allocate funds towards diverse energy sources, encompassing both non‐renewable and renewable options. A reciprocal relationship was discovered between FDI and non‐renewable energy sources. In contrast, in another study Ahmad et al.^[^ ^34^ ^]^ unveiled that the increasing FDI contributes to enhancing the utilization of sustained sources of energy which shows a positive liaison between FDI and renewable consumption of energy. However, the work of Kilicarslan^[^ ^35^ ^]^ and Lin et al.^[^ ^36^ ^]^ did not get the direct link between FDI and renewable energy sources and they also negated this positive relationship. While the work of Doytch and Narayan^[^ ^37^ ^]^ explored that FDI discourages the consumption of lethal energy sources. Moreover, a U‐shaped link exists between FDI and the sustained energy utilization sources.

The whole world is interlinked in different aspects and globalization in terms of business is one of them. When an issue is globalized then it does not matter how big it is, but it attracts the attention of the entire world. After getting recognition worldwide, it changes rapidly. Globalization interacts and interlinks all countries in the aspect of economically, socially, and politically. It has also affected human lives and economies. The term “ongoing economic globalization” expands the welfare and betterment of an open economy in general through the source of FDI, cash flow, and international trade.^[^ ^38^ ^]^ The study of Gozgor et al.^[^ ^39^ ^]^ explained that the term globalization minimizes the information asymmetry and makes things more clear which boosts promoting the mechanism of renewable energy. They also found a positive and significant link between globalization and renewable energy sources.

The study of Shahbaz et al.^[^ ^40^ ^]^ found a positive link between globalization and REC which means that the term globalization minimizes the utilization of energy through the channel of energy efficiency and low CO2 emitting technology. In another study of Shahbaz, Lahiani, et al.^[^ ^41^ ^]^ showed that globalization promotes the utilization of renewable energy sources which reveals a positive link. Globalization promotes renewable energy utilization in Ireland and Netherland during 1970–2015.^[^ ^42^ ^]^ However, the work of Destek^[^ ^43^ ^]^ showed an inverse liaison between globalization and the REC. Another study of Destek and Sinha^[^ ^44^ ^]^ described that the utilization of renewable energy is discouraged by different economies while fulfilling massive demand. They further briefed that there is a negative link between usage of renewable sources of energy and globalization. Various developing economies are energetic to globalization and give priority to consuming non‐renewable sources of energy which reveals that there is an inverse link between globalization and renewable utilization of energy.^[^ ^45^ ^]^ The study of Rafindadi and Ozturk^[^ ^46^ ^]^ noted that, In Germany, In Germany, the consumption of renewable energy significantly strengthens economic growth, with a 1% increase in renewable energy usage enhancing economic growth by 0.2194%. Furthermore, a 1% rise in capital investment results in a 1.1320% increase in economic growth.

The existing literature provides insights into the relationship between trade orientation, technological innovation (TI), and the utilization of renewable energy sources. However, there is a notable gap in substantial investigations concerning the connection between trade orientation and TI on renewable energy consumption (REC) within the G‐7 economies. The current research suggests that advancements in technology, characterized by TI, play a significant role in reducing the cost and improving the efficiency of renewable energy production, thus fostering its utilization.

## 3 Data and Methods

This empirical work incorporates secondary data. Data availability determines sample size. The sample includes 138 panel observations and 7 cross‐sections from 2000 to 2019. The sample contains annual data from the G‐7 countries. We have picked G‐7 countries for our analysis for numerous reasons. First, The G‐7 includes the seven most industrialized nations in the world, which conduct substantial trade. These countries contribute 46% to global GDP and utilize 23% of global energy.^[^ ^47^ ^]^ Second, among G‐7 countries, five countries (the US, Japan, the UK, Germany, and France) among the 10 leading energy consumers. All G‐7 countries except Canada have an ecological deficit, meaning they use more natural resources than their population. Third, as a top energy consumer, the US has extensive trade and financial ties with the G‐7. These characteristics give G‐7 panel cross‐sectional dependence, a fundamental assumption of the methodological tool used (i.e., G‐7 countries have complicated economic ties) by considering balanced panel data. The world development indicators (WDI), published by the World Bank, provided information on macroeconomic and environmental aspects, whereas the KOF index provided information on the globalization variable. Data availability statement can be found in supporting information.

The study's reliance on data up to 2019, though five years old, is a deliberate methodological choice grounded in the availability and reliability of comprehensive data. Data for the years beyond 2019, particularly in the context of renewable energy consumption, technological innovation, and trade orientation, are often incomplete or inconsistent, which could compromise the robustness of the analysis. Moreover, the period following 2019 is marked by the global COVID‐19 pandemic which introduced significant disruptions in trade dynamics, industrial activities, and energy consumption patterns. Including this period could confound the results, as the observed effects might reflect pandemic‐induced anomalies rather than structural relationships. By focusing on the pre‐2020 period, the study ensures an analysis of stable, long‐term trends, providing clearer insights into the impacts of technological innovation and trade orientation on renewable energy consumption. Furthermore, the 20‐year timeframe (2000–2019) offers a sufficiently robust dataset to identify these relationships without the influence of short‐term shocks, ensuring that the policy implications are based on normal economic conditions rather than crisis scenarios. Our models' frameworks are described as follows:

(1) $$REC=f\;\left(TECH,EXPO,IMPO,GDP,FD,FDI,GLOB\right)$$

In econometric form, Equation (1) can be written as follows:

(2) $$\begin{array}{ccc} & & REC_{it}=\alpha_{0}+\alpha_{1}TEC_{it}+\alpha_{2}EXPO_{it}+\alpha_{3}IMPO_{it}+\alpha_{4}FD_{it}\\ & & \;\;+\alpha_{5}FDI_{it}+\alpha_{6}GLOB_{it}+\varepsilon_{it} \end{array}$$

where REC is renewable energy consumption, and TECH is TI from G‐7 economies. EXPO and IMPO indicate the export and import of a country. Moreover, GDP, FD, FDI, and GLOB are used as control variables. Moreover, α measures the estimated coefficients of variables (TECH, EXPO, IMPO, GDP, FD, FDI, GLOB). The subscript ε_*i*_ denote to error terms, where i represents cross sections consisting of seven countries. The subscript t represents the period from 2000 to 2019.

### 3.1 Variables

The dependent variable of the study is REC. Renewable energy sources are important for lowering greenhouse gas emissions, especially CO2, which makes up more than 60% of GHGs. Renewable energy is important if the long‐term average rise in global temperature is to stay between 2 and 2.4 °C and the world's goal is to lower CO2 by 2050. The understanding that significant energy use in the form of fossil fuels results from industrialization, globalization, and population increase has pushed the adoption of renewable energy sources.^[^ ^48^ ^]^ For instance, renewable energy is a cost‐effective and technically feasible GHG‐reduction strategy. Renewable energy can help to bring down energy costs, improve air quality, have a good impact on the ecology, and create jobs.^[^ ^49^ ^]^ Utilizing domestic clean energy resources like solar, wind, hydropower, biomass, and geothermal energy can also lower the costs of energy imports.^[^ ^50^, ^51^, ^52^ ^]^ Due to numerous environmental concerns, countries are switching to renewable energy sources. Perhaps, REC can play a vital role in achieving promising progress with more policy support to meet sustainability goals.

Technological innovation, on the other hand, is handled as an independent variable. Undoubtedly, renewable energy technology will persist in bolstering economic growth, as well as promoting social and environmentally sustainable development. It will also enhance the nation's standing and the importance of alternative energy sources. New technologies can now be widely used in the economy because of technological progress. It enables the economy to use less energy while still producing the appropriate level of output. Additionally, the implementation of technology could expedite the acceptance of renewable energy, thereby meeting the increasing energy requirements.^[^ ^2^, ^53^, ^54^ ^]^ Technology makes the energy industry more competitive, lessens the damage that energy use does to the environment, and lowers the cost of energy for consumers. In current analysis, TECH and REC are assumed to be positively related. The volume of imports and exports as a percentage of GDP defines trade orientation in this study. This metric captures the degree to which a country's economy is integrated into the global market. A higher export orientation indicates a robust involvement in international trade, often reflecting a competitive edge in certain industries and a drive for innovation. Conversely, a higher import orientation may indicate dependency on foreign goods and services. By examining these ratios, the study assesses how a nation's trade dynamics influence renewable energy consumption, highlighting the importance of balancing import and export activities to foster sustainable energy practices.

It is true that international trade and the use of renewable energy are related.^[^ ^55^, ^56^ ^]^ Trade can increase REC for numerous reasons. First, an increase in trade of goods necessitates a greater amount of energy, particularly renewable energy, for the production and transportation of these commodities. Second, the cost of renewable energy equipment has been reduced due to economies of scale and technological advancements, prompting firms to explore new markets. This enhances the global accessibility of renewable energy for a broader range of users. Third, international trade can be a vital factor in transferring renewable energy technology and greening the energy industry. When a country imports renewable energy devices and equipment, it transfers technology through trade. We expect positive trade impacts, therefore, trade benefits renewable energy. Trade orientation refers to the strategic focus of an economy on international trade activities, encompassing both exports and imports. It is quantitatively measured by the percentage of exports or imports relative to the total GDP within a given period. In particular, export orientation denotes the extent to which a country emphasizes exporting goods and services to foreign markets while import orientation reflects the degree to which a nation relies on imported goods and services to meet domestic demand.

Control variables include; gross domestic product (GDP) is a common driver of energy consumption since it increases demand for raw materials, technology, labor, and energy solutions.^[^ ^57^ ^]^ Countries typically emphasize industrial productivity over environmental sustainability, resulting in non REC. Financial development (FD) boosts the need for greener energy usage,^[^ ^58^ ^]^ but a quick switch in energy sources may disrupt domestic production. Financial limitations may force them to continue using fossil fuels. Foreign direct investment (FDI) inflows into the host economies can be facilitated by increased profit potential and enhanced economic expansion through technological spillover, which may encourage economies to move towards environmentally friendly energy uses, i.e., renewable energy.^[^ ^39^ ^]^ We postulate that these spillovers also apply to renewable energy. Globalization (GLOB) has the potential to enhance environmental quality and promote sustainable economic growth by means of renewable energy.^[^ ^41^ ^]^ G‐7 countries can focus on how globalization affects the quality of the environment by studying the dynamics of the demand for renewable energy instead of economic development. This is because they may use energy sources that release less carbon dioxide when making things. **Table** **1** lists the variables that were considered in the analysis.

**Table 1:** Variables particulars (Note: “DV” denotes the dependent variable, “IND” the independent variables, and “CV” the control variables. Source: previous studies).

Acronyms	Full name	Role	Measurement	Source
REC	Renewable Energy Consumption	DV	% of total final energy consumption	WDI
TECH	Technological innovations	IND	% of GDP	WDI
EXPO	Export	IND	% of GDP	WDI
IMPO	Import	IND	% of GDP	WDI
FD	Financial development	CV	% of GDP	WDI
FDI	Foreign direct investment	CV	% of GDP	WDI
GLOB	Overall Globalization	CV	Index	KOF

### 3.2 Data Assessment

We initially run the pooled OLS, even though it is predicted that the G‐7 countries will have varying levels of REC and TI, which could lead to high probability of some econometric problems. Prior to estimating variables, we estimate the cross‐section dependence, stationarity, and cointegration. A panel‐based study has CD (cross‐section dependence) concerns. CD data errors might lead to skewed results and misleading information if not addressed. According to several studies,^[^ ^59^ ^]^ the CD test must be performed before testing framework stationarity. This empirical investigation makes use of the Lagrange multiplier (LM) and cross‐sectional^[^ ^60^ ^]^ for checking the CD issue. We test the following equation to estimate the CD.

(3) $$\mathrm{CD}=\sqrt{\frac{2T}{N\left(N-1\right)}\;\left(\sum_{i=0}^{N-1}\sum_{j=i+1}^{N}\rho_{ij}\right)}\;$$

Here, *T* denotes the passage of time, CD indicates cross‐sectional dependence, and N denotes cross‐sectional correlation. The cross‐sectional correlation of errors between *i* and *j* is further defined asρ_*ij*_. We employ the following equation (LM test) to investigate the CD:

(4) $$y_{it}=\alpha_{it}+\beta_{i}x_{it}+\mu_{it}$$

In this case, *i* represents the cross‐sections and *t* represents time. For both methodologies, the alternative hypothesis explains that cross‐sections are dependent on one another, while the null hypothesis suggests that the cross‐sections between the variables are independent. **Table** **2** displays the statistical analysis for CD issues. The empirical results indicate that CD exists. It demonstrates the strong dependence between all G‐7 countries.

**Table 2:** Cross sectional dependence approach (Note: The existence of CD is inferred from the significant probability. Source: own estimation).

	t‐statistic	Prob.
Breusch‐Pagan LM	131.574	0.000
Pesaran scaled LM	15.982	0.000
Pesaran CD	7.851	0.000

In the presence of CD issue, we estimate the stationarity by employing the second‐generation unit root test. The empirical analysis shown in **Table** **3** reveals that most series appear not to be stationary at level according to both criteria. However, at the first difference, REC, TECH, EXPO, IMPO, FD, and GLOB are stationary. We found that all variables are stationary after obtaining the first series difference.

**Table 3:** Panel unit root approach (Note: see the abbreviation list. Source: own estimation).

	CADF				CIPS			
	*I*(0)		*I*(1)		*I*(0)		*I*(1)	
Variables	Stat.	Prob.	Stat.	Prob.	Stat.	Prob.	Stat.	Prob.
REC	1.767	0.961	−5.657	0.000	4.237	1.000	−4.521	0.000
TECH	−1.66	0.048	−6.387	0.000	−0.257	0.399	−5.420	0.000
EXPO	2.397	0.992	−1.565	0.059	3.153	0.999	−3.711	0.000
IMPO	−1.934	0.027	−6.257	0.000	0.181	0.572	−4.750	0.000
GDP	−6.117	0.000	–	–	−5.024	0.000	–	–
FD	−0.193	0.424	2.687	0.096	−0.450	0.326	−3.237	0.001
FDI	−1.028	0.152	–	–	−3.196	0.001	–	–
GLOB	−1.071	0.142	−8.052	0.000	1.714	0.957	−6.765	0.000

After the stationarity check, we apply a cointegration test. Westerlund^[^ ^61^ ^]^ cointegration tests explore the long‐run relationships between variables. These methods use the null hypothesis (Ho) to explain no cross‐section cointegration. The cointegration results are reported in **Table** **4**. The statistics of prob‐value reject the null hypothesis, indicating that cointegration exists between variables.

**Table 4:** Cointegration approach (Note: The significant result accepts the H_0_ of no cointegration. Source: own estimation).

Test	Statistic	Prob.
Westerlund cointegration test		
Variance ratio	2.286	0.011

The possible remedies in case of cross‐sectional dependence are GLS‐generalized least square and PCSE‐panel corrected standard error. In the case of our analysis, N<T so best suited estimation technique is PCSE (panel corrected standard error). As there is no cointegration, this study adopted the pooled mean group (PMG) methodology. The PMG method was first introduced by Pesaran et al.^[^ ^62^ ^]^ The PMG technique assumes coefficients are homogeneous, but in the short‐term, they can change between cross‐sections. Panel ARDL/PMG models specifically tackle issues related to cross‐sectional dependence, serial autocorrelation, and cross‐sectional heteroscedasticity. The PMG technique provides estimates of the Error Correction Term or Speed of Adjustment, which is an essential element of PMG. The PMG/ARDL paradigm allows for the utilization of many variables to determine a single variable lag. The Akaike Information Criteria (AIC) is most suitable for selecting the lag order, handling constrained data, and simplifying models.

In the current study, G7 is a group and we are unable to use the structural break test as the study aims to check the impact in the group. Moreover, the study's emphasis on employing panel data econometric techniques such as Panel EGLS and ARDL models have obviated the need for separate structural break tests. These techniques are adept at capturing longitudinal dynamics and cross‐sectional variations within the data, potentially encompassing shifts in relationships over time. Given the scope and objectives of the study, which likely prioritized understanding long‐term trends and overarching patterns across G‐7 economies, we favored a comprehensive analytical approach over the intricacies introduced by structural break tests. Lastly, considerations regarding the complexity of interpretation and adherence to statistical assumptions could have influenced the decision‐making process. By opting for a more straightforward analytical framework, we aimed to ensure clarity and robustness in their findings, ultimately aligning with the study's overarching objectives and methodological preferences.

## 4 Empirical Analysis

In **Table** **5**, the mean value of REC is 10.482, indicating the percentage consumption of renewable energy over total consumption of energy. The mean value of TEC is 2.206, illustrating the percentage of research and development expenditures as compared to total GDP. Moreover, the mean value of TECH reflects the intensity of TI within a country. The EXPO has a mean value of 26.372, indicating the percentage volume of export of goods and services as compared to total GDP. Meanwhile, the IMPO has a mean value of 26.160 of total GDP. In addition to the intensity of export and import, it can also be viewed that EXPO is slightly higher than IMPO, indicating the more focus of G‐7 nations on export orientation. The average value of GDP (economic growth) in G‐7 economies is 1.498 with the highest value of 6.868 (for Canada in 2007) and a minimum value of ‐5.693 (for Germany in 2009). The mean value of FD (98.226) implies the percentage volume of funds extended by banks to the private sector of an economy. The volume of FDI inflow is 2.130 percent of GDP while the mean value of globalization is 82.355 which is an index value of globalization trend in G‐7 economies. In addition, **Table** **6** presents the correlation values for the variables of the study.

**Table 5:** Descriptive approach (Note: see the abbreviation list. Source: own estimation).

Variables	Mean	Median	Std. dev.	Max.	Min.
REC	10.482	9.055	0.062	22.690	0.850
TECH	2.206	2.185	0.065	3.367	1.040
EXPO	26.372	27.894	0.102	47.301	9.035
IMPO	26.160	27.762	0.082	41.133	9.089
GDP	1.498	1.688	0.191	6.868	−5.693
FD	98.226	96.113	0.310	190.943	48.974
FDI	2.130	1.714	0.021	11.929	−0.863
GLOB	82.355	83.000	0.052	90.000	67.000

**Table 6:** Correlation analysis (Note: see the abbreviation list. Source: own estimation).

Variables	REC	TECH	EXPO	IMPO	GDP	FD	FDI	GLOB
REC	1.000							
TECH	−0.236	1.000						
EXPO	0.555	−0.281	1.000					
IMPO	0.575	−0.391	0.666	1.000				
GDP	0.128	0.034	0.097	0.126	1.000			
FD	0.040	−0.311	0.289	0.349	−0.013	1.000		
FDI	0.045	−0.296	0.241	0.316	0.325	0.259	1.000	
GLOB	0.257	−0.371	0.669	0.783	0.084	0.275	0.373	1.000

### 4.1 Regression Analysis

In **Table** **7**, we have presented the regression outcomes of panel EGLS (estimated generalized least square) and ARDL (autoregressive distributed lag) models. The coefficient value of TI is 0.062, which is significant at a 5% level and indicates the increase in REC due to a one‐unit change in TI. This positive significant impact of TI on renewable energy remains consistent even in the case of the ARDL model. The coefficient value of export orientation is 0.346 while the coefficient value of import orientation is 1.190. These values predict the significant positive effect of export orientation while a significant negative effect of import orientation on renewable energy. Moreover, the coefficient values explain that a one‐unit change in export orientation can uplift the REC by 34.6% while import orientation impedes the REC by 119.0% relatively. Economic growth carries a negative significant coefficient value of ‐0.229, indicating the inverse relationship between GDP and REC. Similarly, financial development has a negative significant coefficient value of ‐0.029 in the short run (EGLS produces the results in the short run), while a significant positive coefficient value of 0.136 in the long run (ARDL produces the results in the long run). Similarly, FDI inflow has a negative significant value of ‐0.249 in the short run while a significant positive coefficient value of 0.672 in the long run. Nonetheless, globalization has negative significant coefficient values of ‐0.585, and ‐0.503 both in the short‐run and long‐run relatively. The estimated coefficient values of explanatory variables predict the direction and strength of association of underlying explanatory variables with REC.

**Table 7:** Trade orientation, technological innovation, and renewable energy (Note: ***, **, * denote the significance at 1%, 5%, and 10% relatively. Source: own estimation).

Renewable energy as a dependent variable (testing Equation 2)				
	Panel EGLS		ARDL	
Variables	Coefficients	Stand. Error	Coefficients	Stand. error
Constant	40.497***	5.577	–	–
Technological innovation	0.062**	0.0322	3.462***	1.134
Export orientation	0.346***	0.103	0.612***	0.041
Import orientation	−1.190***	0.164	−0.388***	0.037
Economic growth	−0.229**	0.121	−0.984***	0.106
Financial development	−0.029***	0.006	0.136***	0.007
Foreign direct investment	−0.249***	0.083	0.672***	0.138
Globalization	−0.585***	0.080	−0.503***	0.103
*Adjusted R‐square*		0.833	0.396	
*S.E. of regression*		0.186	0.043	
*Prob. (f‐statistics)*		0.000	−	

## 5 Discussion on Results

This study aims to find out the impact of TI and trade orientation on REC. To achieve the aim, we employ the panel EGLS and ARDL models and report the results in Table 7. Based on estimated coefficient values, TI exerts a positive significant impact on REC. Industrialists always require a strong motivation to transform their fuel needs from outdated sources of energy to REC. In this essence, TI allows such transformation by boosting the production of new machinery that is less dependent on fossil fuel energy and enhances energy efficiency, and product quality.^[^ ^1^ ^]^ All these factors urge the industrial managers to follow the REC as fuel inputs. Meanwhile, TI integrates favorable competition both in terms of product quality and cost efficiency which further compels the industrialists to transform their production based on non‐renewable to renewable sources of energy.^[^ ^15^ ^]^ Supporting this, Solarin et al.^[^ ^14^ ^]^ have argued that TI is mandatory to boost the REC, stemming from energy efficiency, reduction of costs, and a strong motivation for industrial producers to transform their production units from fossil fuels to REC. The strong TI turns the attitude of industrial managers from consumption of cost‐intensive sources, i.e., fossil fuels to renewable sources. Moreover, the TI mitigates the extant stress of environmental sustainability on enterprises by allowing industrialists to follow renewable sources as fuel inputs.^[^ ^16^ ^]^

As the analysis shows, the export orientation leads to enhancing the REC whilst import orientation declines the REC. Export‐oriented countries are more concerned regarding product quality and green productivity and therefore utilize more REC as compared to fossil fuel energy. In this regard, Chen^[^ ^19^ ^]^ empirically supports this notion by exploring the link between the export and utilization of renewable energy as fuel inputs. The empirical findings of his study asserted that export orientation compels the countries to utilize more renewable energy as compared to fossil fuel energy. The countries with export orientation try to mitigate the production costs and better product quality to surpass the international price and quality competition. In this essence, the utilization of renewable energy helps to achieve such goals as REC is an economical source of energy and has positive outcomes regarding product quality.^[^ ^22^ ^]^ Moreover, the REC allows the industries to claim green productivity which has a positive spillover effect regarding the increment in export volume. On the other hand, the import orientation deepens the consumption of fossil fuels due to more focus on imported goods. A country with a high import orientation is unable to adopt the modern production systems and thus massively utilize fossil fuels as fuel inputs. Bento and Moutinho^[^ ^21^ ^]^ commented that international trade enhances the lethal gas emissions due to more inflow of industrial goods. Import orientation impedes domestic industrial competition due to more dependence on imported goods and thus industries keep utilizing outdated sources of energy. In contrast, the export orientation augments the competition both at the domestic and international level and therefore managers are more concerned about costs, quality, and even green productivity. Thus, it can be asserted that the export orientation encourages the REC while import orientation defers the consumption of renewable energy.

Explaining the effect of control variables on REC, the economic growth shows a negative relationship with REC. With the function of more industrial activities, this negative effect of economic growth can be explained as higher economic growth requires the exploration of such industrial activities, e.g., heavy production of industrial products that require more consumption of fossil fuel energy. Hence, economic growth discourages the REC and makes REC a less preferred source of energy. Initially, the countries are more concerned about economic growth rather than environmental sustainability and achieve this motive of economic success by exploiting the more natural resources which harness the consumption of REC.^[^ ^29^, ^63^ ^]^ The famous EKC model also supports this notion. Financial development has a negative impact in the short run while a positive impact in the long run upon REC. In the short run, the developed financial sector enhances the consumption of fossil fuel energy due to the availability of funds which induces a positive influence on cost‐intensive energy sources, i.e., fossil fuel energy sources.^[^ ^24^ ^]^ However, in the long run, the developed financial sector reduces the pollution emissions by encouraging the industrial sector to transform their fuel needs from fossil fuel to REC sources and by offering green funds.^[^ ^64^, ^65^, ^66^, ^67^ ^]^

Similarly, the inflow of FDI asserts a positive effect on REC in the short run while a negative impact in the long run. In view of boosting the inflow of FDI, the host country may allow the foreign investors to keep growing even by utilizing fossil fuel energy. This specific relationship between foreign investment and REC can be supported by the famous pollution heaven hypothesis, which argues the positive relationship between FDI and pollution emissions, stemming from ore fossil fuel consumption. However, this pollution‐intensive consumption of fuel is discouraged by applying both environmental regulations and quality checks by the local government in the long run. Moreover, the pollution hallo hypothesis reveals that foreign investors utilize the REC to enhance their market reputation when the aim is to sustain it for the long run.^[^ ^35^, ^37^ ^]^ Thus, it can be asserted that foreign entrepreneurs utilize more fossil energy in the short run while more REC in the long run. Lastly, globalization shows an inverse relationship with REC. In contrast to existing literature, suggesting the positive impact of globalization on REC.^[^ ^40^, ^41^, ^42^ ^]^ Moreover, this specific impact can be explained as globalization enhancing the interaction among countries regarding the trade and thus can enhance the competition, which further proceeds to enhancing the production volume even by utilizing more fossil fuel energy. In summary, the TI and export orientation have positive whilst import orientation produces negative links with REC.

The findings of this study offer robust insights for policymakers, with several strengths and some limitations. A key strength is the use of advanced econometric techniques (Panel EGLS and ARDL models) that account for cross‐section dependence and stationarity issues, ensuring the reliability of the results. The positive correlation between technological innovation and export orientation with renewable energy consumption provides clear directives for policy initiatives focused on innovation and trade enhancement. However, a potential weakness lies in the study's exclusive focus on G‐7 economies, which may limit the generalizability of the findings to other countries with different economic structures. Additionally, the negative impact of import orientation on renewable energy consumption warrants further investigation to understand underlying mechanisms and mitigate adverse effects. Despite these limitations, the study offers valuable guidance for promoting sustainable energy policies.

The findings of this study have important implications for advancing the Sustainable Development Goals (SDGs), particularly SDG 7 (Affordable and Clean Energy) and SDG 13 (Climate Action). By demonstrating the positive impact of technological innovation and export orientation on renewable energy consumption, the study highlights actionable pathways to enhance global energy sustainability. Technological advancements directly contribute to improving energy efficiency, reducing greenhouse gas emissions, and making renewable energy more affordable and accessible, aligning with SDG 7. Similarly, the promotion of export‐oriented trade strategies for renewable energy technologies fosters international cooperation and the diffusion of clean energy solutions, advancing SDG 13 by supporting global efforts to combat climate change. Policymakers are encouraged to leverage these insights by fostering innovation ecosystems, increasing investments in green technologies, and formulating trade policies that prioritize renewable energy exports, ensuring a sustainable and equitable energy future.

## 6 Conclusion and Policy Implications

This empirical analysis subjects to unveiled the role of TI and trade orientation on REC. To ascertain the regression among variables, we sample the G‐7 economies and employ the panel EGLS and ARDL models to disseminate the regression. The statistical results revealed that TI has a direct effect on REC as it encourages the industrial sector to transform their energy needs from ancient sources of energy to renewable sources. We further found a positive correlation between export orientation and REC. More focus on export orientation is deemed to be quality checks and cost efficiency that can be achieved by utilizing renewable sources. Nonetheless, the import orientation asserted a negative impact on REC. More trade activities specifically import activities discourage the consumption of REC due to low integration of product competition at a domestic level. In addition, the current analysis vowed the negative effect of all control variables including economic growth, financial development, foreign investment, and globalization in the short run. However, financial development and foreign investment asserted a positive effect on REC in long run. The estimated coefficient signs of explanatory variables suggested accepting the theoretical suggestions developed in the current study. In addition, the ongoing empirical analysis provided robust evidence on the pollution halo hypothesis, pollution haven hypothesis, and EKC model in the G‐7 case.

The findings of this study align closely with the research objective and problem which aim to explore the impact of technological innovation and trade orientation on REC in G‐7 economies. The study successfully demonstrates that technological innovation and export orientation significantly and positively influence renewable energy consumption, thus addressing the core research problem of identifying the drivers of renewable energy uptake. Moreover, the negative impact of import orientation offers a critical perspective on the potential hindrances to renewable energy growth. These insights not only fulfill the research objective of understanding the dynamics influencing renewable energy consumption but also provide actionable guidance for policymakers. The study effectively positions its findings within the broader context of promoting environmental sustainability and reducing pollution, thereby achieving its end purpose of contributing valuable knowledge to the field of energy economics and policy‐making.

The study highlights several key implications for policymakers and industry leaders. First, it suggests that prioritizing technological innovation and export‐oriented trade policies can significantly enhance REC. Governments should invest in research and development to drive advancements in renewable energy technologies, thereby boosting REC. Additionally, creating financial and regulatory incentives for industries to adopt and develop these technologies will encourage their widespread use. Adjusting trade strategies to reduce reliance on energy imports and focus on expanding exports of renewable energy technologies can further positively impact REC. By aligning national policies with these findings, countries can better achieve their sustainability goals and contribute to global efforts in reducing pollution. This study emphasizes the need for integrating technological and trade considerations into long‐term energy planning to ensure sustained growth in renewable energy consumption.

### 6.1 Policy Implications

Based on the empirical reasoning of the current study, the important policy layout is to more focus on TI at the country level. By promoting more consumption of renewable energy, such innovation can produce positive outcomes regarding environmental sustainability. Thus, policy officials should encourage the exploration of R & D activities by offering subsidies, direct funds, and ethical support. Similarly, the export orientation can also be utilized as a policy tool to enhance the consumption of REC. Both factors, i.e., export orientation and REC have positive interchangeable effects on each other. More dependence can be used as a facilitator of export enrichment and export orientation can be used as a catalyst to promote the REC, which further has many positive outcomes in the form of environmental sustainability and industrial growth. This study shows the positive effect of foreign investment and financial development on REC in long run. Thus, the policy analysts should consider such factors as a long‐term strategy to encourage the industrial sectors on more REC. Moreover, it is suggested to policy analysts from G‐7 economies to revisit the attached energy policies with economic growth, financial development, foreign investment, and globalization even in the short run. The policy officials should align these factors with the consumption of green energy, i.e., renewable energy.

This study uncovers several nuanced insights that are crucial yet often overlooked in the discourse on REC. One hidden fact is the significant negative impact of import orientation on renewable energy use, suggesting that reliance on imported goods may stifle domestic renewable energy initiatives. Another subtle revelation is the interplay between technological innovation and export orientation, highlighting how technological advancements can enhance a country's competitive edge in international markets while promoting clean energy consumption. Philosophically, this study addresses a key gap in understanding how macroeconomic factors like trade orientation and technological progress shape renewable energy dynamics. By empirically linking these elements, the study provides a comprehensive framework that underscores the interconnectedness of economic policies and environmental sustainability, paving the way for more informed and holistic policy‐making in the future.

### 6.2 Limitations and Future Research

While the study provides valuable insights across G‐7 economies, several limitations warrant acknowledgment. Future research could address these limitations by incorporating more granular data sources, such as firm‐level or regional data, to elucidate context‐specific dynamics. Additionally, employing advanced econometric techniques that account for structural breaks could enhance the robustness of findings. Furthermore, exploring the role of additional factors, such as policy interventions and socio‐cultural influences, could provide a more comprehensive understanding of the determinants of renewable energy consumption. Overall, addressing these limitations and expanding the scope of analysis can enrich understanding of the factors shaping renewable energy [Supplementary-material gch21678-supitem-0001] transitions in G‐7 economies.

## Conflict of Interest

The authors declare no conflict of interest.

## Supporting information

Supporting Information

## Data Availability

The data that support the findings of this study are available from the corresponding author upon reasonable request.
